# The whole genome analysis of four Orf virus strains from Europe and South America

**DOI:** 10.1093/ve/veag004

**Published:** 2026-01-28

**Authors:** Marco Cacciabue, Laura C Lozano Calderón, Javier Moleres, Irache Echeverría, Lorena de Pablo, Idoia Glaria, Guido König, Andrea Peralta, Ramsés Reina

**Affiliations:** Departamento de Ciencias Básicas, Universidad Nacional de Luján, Luján, Buenos Aires, Argentina; Instituto de Agrobiotecnología y Biología Molecular (IABiMo), Instituto Nacional de Tecnología Agropecuaria (INTA), Consejo Nacional de Investigaciones Científicas y Técnicas (CONICET), Hurlingham, Buenos Aires, Argentina; Instituto de Agrobiotecnología y Biología Molecular (IABiMo), Instituto Nacional de Tecnología Agropecuaria (INTA), Consejo Nacional de Investigaciones Científicas y Técnicas (CONICET), Hurlingham, Buenos Aires, Argentina; Instituto de Agrobiotecnología, (CSIC-Gobierno de Navarra), Avda. Pamplona 123, 31192 Mutilva, Navarra, Spain; Instituto de Agrobiotecnología, (CSIC-Gobierno de Navarra), Avda. Pamplona 123, 31192 Mutilva, Navarra, Spain; Dpto. de Agronomía, Biotecnología y Alimentación, Universidad Pública de Navarra. Campus Arrosadía, 31006, Pamplona, Navarra, Spain; Instituto de Agrobiotecnología, (CSIC-Gobierno de Navarra), Avda. Pamplona 123, 31192 Mutilva, Navarra, Spain; Instituto de Agrobiotecnología, (CSIC-Gobierno de Navarra), Avda. Pamplona 123, 31192 Mutilva, Navarra, Spain; Instituto de Agrobiotecnología y Biología Molecular (IABiMo), Instituto Nacional de Tecnología Agropecuaria (INTA), Consejo Nacional de Investigaciones Científicas y Técnicas (CONICET), Hurlingham, Buenos Aires, Argentina; Instituto de Agrobiotecnología y Biología Molecular (IABiMo), Instituto Nacional de Tecnología Agropecuaria (INTA), Consejo Nacional de Investigaciones Científicas y Técnicas (CONICET), Hurlingham, Buenos Aires, Argentina; Instituto de Agrobiotecnología, (CSIC-Gobierno de Navarra), Avda. Pamplona 123, 31192 Mutilva, Navarra, Spain

**Keywords:** parapoxvirus, ORFV, phylogenetic analysis, genomic sequencing

## Abstract

Orf virus (ORFV) is the etiological agent of Contagious Ecthyma, a global disease that mainly affects sheep, goats, wild ruminants, and humans. Here, we determined the complete genome sequence of two ORFV strains from Spain (NAV and ARA) and two from Argentina (HRE and CHB), representing the first report from the Iberian Peninsula and from South America. The assembled genomes of the ARA, CHB, HRE, and NAV strains of ORFV were found to be 137 891 , 137 160, 137 340, and 137 214 bp long, respectively, each contained 132 genes, and all showed high amino-acid identity and similar lengths compared to the reference strain NZ2. We performed a microsatellite analysis to identify molecular signatures associated with host species (sheep or goat). In addition, the analysis of 32 selected genes showed that the median nucleotide substitution rate for the worldwide cluster 3 that includes the four samples described in this study, was 2.6 x10^−5^ subs/site/year (9.2x10^−8^—6.7x10^−5^—95% HPD) placing the TMRCA (median divergence time) around 1730. Genetic characterization of ORFV strains not only allows epidemiological studies but also represents the first step towards developing molecular tools for diagnostics and vaccines.

## Introduction

Contagious Ecthyma (CE), also known as Orf, contagious pustular stomatitis or contagious pustular dermatitis, is a worldwide-distributed viral skin disease affecting primarily goats, sheep, and wild ruminants, but also humans causing self-limiting painful pustular lesions on fingers and hands (McElroy and Bassett 2007). CE is caused by Orf virus (ORFV), a member of the family *Poxviridae*, and the most prevalent within the Parapoxvirus (PPV) genus, which also includes bovine papular stomatitis virus (BPSV), pseudocowpoxvirus (PCPV), PPV of red deer in New Zealand (PVNZ) and PPV of the grey seal (Nettleton *et al.* 1995). ORFV infects epithelial cells causing severe proliferative dermatitis, which evolves from macules, papules, pustules to scabs, and fissured crusts (Nandi *et al.* 2011). Lesions are commonly found around the lips, mouth muzzle, nostrils, teats, and oral mucosa, but can also be found in the oesophagus, hooves, reproductive organs, rumen, or respiratory tract (Zhao *et al.* 2010). Albeit lesions usually resolve in 1 to 2 months, reinfection is commonly observed (McKeever *et al.* 1988), regardless the administration of live-attenuated vaccines. In some countries, ORFV vaccines produced by multiple passages in cell cultures, achieving attenuation, are marketed. Despite the potential advantages of this type of vaccine, several drawbacks have been reported, ranging from virulence reversion, gene loss, vaccination failure, or contamination with other microorganisms during production (Asín *et al.* 2021, Buddle and Pulford 1984, Cottone *et al.* 1998, Jorge and Dellagostin 2017).

ORFV infection is endemic in most countries with sheep/goat-raising industries (Robinson and Balassu 1981). Affected animals, mainly young lambs, dramatically reduce their food intake due to the occurrence of painful lesions, causing transient impairment in daily weight gain, and therefore, to important economic losses in farms with recurrent outbreaks. Noteworthy, the morbidity rate often reaches 100%, and despite the mortality rate is usually low, secondary bacterial infections, such as, pododermatitis and mastitis (Burriel 1997), further increase sanitary costs (Haig and Mercer 1998).

The ORFV genome consists of 132 putative genes distributed along a ~ 135kbp linear double-stranded DNA (Delhon et al. 2004) with an unusually high GC content (~64%) under positive selection (Sahu et al. 2020). Recently, recombination has been suggested as the main force driving genetic evolution and ORFV virus diversity (Sahu et al. 2020), which may jeopardize vaccination strategies. Strikingly, despite worldwide distribution and increasing reports of Orf outbreaks, relatively few strains have been isolated, a small proportion have been completely sequenced and little genetic information of the related strains is available. There are 48 whole genome sequences publicly available, derived from sheep, goats, wild ruminants, and one human sample. For our analyses we selected 44 genomes from sheep and goats, including isolates from China, Malaysia, North America, Germany, France, Italy, India and New Zealand (see Table 1).

**Table 1:** Strains of ORFV, PCPV, and BSPV used in this study.

Strain	Country of isolation	Year of isolation	Host	Accesion number	Reference
NZ2	New Zeland	1982	Sheep	DQ184476	Mercer et al. 2006
ARA	Spain	2014	Sheep	ON805833	This study
CHB	Argentina	2016	Sheep	ON805830	This study
HRE	Argentina	2015	Sheep	ON805831	This study
NAV	Spain	2017	Sheep	ON805832	This study
NA1	China	2011	Sheep	KF234407	Li *et al* 2015
NA17	China	2016	Goat	MG674916	Zhong *et al.* 2019
SY17	China	2016	Sheep	MG712417	Zhong *et al.* 2019
HN3	China	2012	Sheep	KY053526	Chen et al. 2017
D1701	Germany	2011	Sheep	HM133903	McGuire *et al* 2012
SA00	USA	2000	Goat	AY386264	Hosamani *et al* 2009
IA82	USA	1982	Sheep	AY386263	Hosamani *et al* 2009
SJ1	China	2012	Goat	KP010356	Chi *et al* 2005
NP	China	2011	Goat	KP010355	Chi *et al* 2005
GO	China	2012	Goat	KP010354	Chi *et al* 2005
MP	India	2017	Goat	MT332357	Sahu et al. 2020
TVL	USA	2019	Sheep	MN454854	Heare *et al* 2020
YX	China	2012	Goat	KP010353	Chi *et al* 2005
VR634	-	1963	Human (Cow)	GQ329670.1	Hautaniemi et al. 2010
CL18	China	2018	Sheep	MN648219.1	Zhou et al. 2022
F00.120R	Finland	2000	Reindeer	GQ329669.1	Hautaniemi et al. 2010
S6	Italy	2020	Sheep	ON691519	Coradduzza et al. 2022
S10	Italy	2020	Sheep	ON691520	Coradduzza et al. 2022
S15	Italy	2020	Goat	ON691521	Coradduzza et al. 2022
S19	Italy	2021	Sheep	ON691522	Coradduzza et al. 2022
S21	Italy	2017	Sheep	ON691523	Coradduzza et al. 2022
S27	Italy	2019	Sheep	ON691524	Coradduzza et al. 2022
S30	Italy	2019	Goat	ON691525	Coradduzza et al. 2022
HSN_20	Malaysia	2018	Goat	MW537048	Mangga et al. 2022
HSN_22	Malaysia	2019	Goat	OP562382	Mangga et al. 2022
SC	China	2019	Sheep	ON932451	Du et al. 2023
SC1	China	2021	Sheep	ON932452	Du et al. 2023
O1V	China	2024	Sheep	PQ374835	Zhang *et al* 2024
O2W	China	2024	Sheep	PQ374836	Zhang *et al* 2024
CL24	China	2024	Sheep	PV126639	Lv et al. 2025
PA1	Pakistan	2023	Sheep	PP911590.1	not published
MOR20	Morocco	2020	Sheep	PQ685033.1	Elkarhat *et al* 2025
FX10	China	2012	Goat	PP943425.1	Jing et al. 2025
FX17	China	2012	Goat	PP943426.1	Jing et al. 2025
FX86	China	2012	Goat	PP943427.1	Jing et al. 2025
S193	Cuba	2008	Sheep	OR637323.1	Coradduzza et al. 2024
S194	Cuba	2008	Sheep	OR637324.1	Coradduzza et al. 2024
S195	Cuba	2007	Goat	OR637325.1	Coradduzza et al. 2024
Mukteswar_p9	India	2005	Goat	ON380499.1	Kumar *et al* 2021
Mukteswar_p50	India	2005	Goat	ON380500.1	Kumar *et al* 2021
nm-W	China	2020	Goat	OP151442.1	not published
B029	Germany	1996	Human (Sheep)	KF837136.1	Friederichs et al. 2014
Mx08	Canada	2015	Muskoxen	SRR9974556	Dalton et al. 2024
Mx347	Canada	2015	Muskoxen	SRR9974557	Dalton et al. 2024
Mx444	Canada	2017	Muskoxen	SRR9974558	Dalton et al. 2024

Phylogenetic studies usually rely on single genes, being *orf011* (B2L) followed by *orf020* (VIR), *orf132* (VEGF), and *orf059* (F1L) the most reported in the literature (Guo et al. 2004, Hosamani et al. 2006, Chan et al. 2007). According to VEGF alleles (*orf132*), a classification into NZ2- and NZ7-like strains was proposed (Mercer et al. 2006). More recently, according to *orf011* and *orf020* genes, a classification attending to animal species, sheep or goat has been proposed (Velazquez-Salinas et al. 2018; Karki et al. 2019). However, depending on the selection pressures affecting different genes, evolution inferences may suffer from inaccuracy when considering individual or concatenated genes (Yao et al. 2020).

Microsatellites, also known as simple sequence repeats (SSR), are 1-6 bp unit repeated ubiquitously in the genome of eukaryotes, prokaryotes, and viruses (Davis et al. 1999, Tóth et al. 2000, Mrázek et al. 2007, Alam et al. 2013). Compound microsatellites (cSSR) are composed of two or more individual SSRs directly adjacent to each other, for example, (CAG)n-Xn-(TA)n. Some studies have proposed the use of SSR and cSSR for the characterization of viral strains (Walker et al. 2001, Sahu et al. 2020).

In this study, we analysed four different CE outbreaks reported in South America and Europe. ORFV presence was confirmed in all animals by performing specific PCR amplification of *orf045* and *orf011* genes in lesions. Isolation was attempted in different ovine primary cells using scabs and replication was assessed by molecular methods. Complete genome sequences were obtained by NGS methods and analysed regarding genome structure, SSRs, recombination events, evolutionary rate, and phylogenetic inferences.

Genetic characterization of ORFV circulating strains may shed light on the molecular epidemiology that underlies ORFV infection. Deciphering genome content and diversity is a first step towards the development of new detection and prevention tools against CE.

## Material and methods

### Sheep flocks and tissue collection

Spanish flocks from Navarra (42° 49′ 00″ N 1° 39′ 00″ W) and Aragón (41° 39′ 23″ N 0° 52′ 36″ W) under routine veterinary surveillance, were included in the study after reporting two outbreaks of CE in April and May 2017, respectively, (samples NAV and ARA, respectively).

Argentine samples were collected in Huinca Renancó (34° 50′22″ S 64° 22′19″ W) province of Córdoba (sample HRE) and Chacabuco (34° 38′31”S 60° 28′17 “W) province of Buenos Aires (sample CHB) in 2015 and 2016, respectively. In both cases, shepherds did not report previous cases of CE, with the difference that the CHB flock incorporates new animals every year, while HRE is a closed establishment.

Scabs were excised from animals during routine diagnosis by veterinarians, using scalped blades and tweezers, maintained refrigerated until shipment to the respective laboratory and then stored at −80 °C for further analyses. Therefore, ethical approval was not required for reporting these cases.

### Virus purification and DNA extraction

Scabs were thoroughly homogenized in liquid nitrogen and 20 mg of the resulting tissue powder was placed in a microcentrifuge tube and incubated with lysis buffer (100 mM Tris–HCl pH 7.5, 12.5 mM ethylenediaminetetraacetic acid (EDTA), 150 mM NaCl, 0.5% Sodium dodecyl sulfate (SDS)) and proteinase K at 56 °C in a water bath, until complete tissue lysis. DNA was then extracted, according to the manufacturer’s instructions (EZNA DNA tissue kit) and stored at −20 °C.

For ORFV detection, PCRs targeting *045* and *011* genes were carried out using previously described methods (Kottaridi et al. 2006).

### Sample preparation for MiSeq sequencing

In order to purify viral particles from the scabs, a previously developed protocol (Zwartouw et al. 1962) was used with some modifications:

For Argentine samples, 500 mg of scab material were macerated in a pestle under liquid nitrogen until a homogeneous powder was obtained. A 30% suspension (weight/volume) was prepared in TMN buffer (10 mM Tris–HCl pH 7.5; 1.5 mM MgCl_2_; 10 mM NaCl).

In the case of the Spanish samples, given the limited starting material (< 50 mg of scab), viral amplification was carried out in ovine epithelial cell culture (Eov). The Eov cell culture was derived from a skin biopsy of a healthy adult sheep at the Virology Institute (INTA, Argentina) in 2017. It was analysed to rule out adventitious viruses at the same institute. The number of passages (eight) before the culture entered senescence and ceased to thrive was determined. Passages 4 and 5 were evaluated for ORFV multiplication. Initially, half of the scab maceration was inoculated into one well of a 6-well plate, and the characteristic cytopathic effect of ORFV was observed at Day 3 post-infection. The cells and supernatants were harvested and subjected to three cycles of freezing and thawing. This first passage was used to infect a T25 flask, until the cytopathic effect was evident. The cells and supernatant from the two passages were combined with the second half of the scab sample for further purification along with the Argentine samples.

Samples were freeze-thawed at −80 °C once and sonicated for three cycles of two minutes each, in bath (Elmasonic, sweep mode). Then, the suspension was centrifuged at 2000 x g for 10 min at 4 °C. Clarified supernatant was loaded onto a 30% sucrose cushion (w/w in TMN buffer) and pelleted at 39 000 g for 30 min (Beckman rotor 70Ti). Pellets were resuspended in 2 ml of TMN buffer and further purified on a sucrose cushion (30%–60%) by centrifuging at 12 °C in a swinging rotor (SW41) at 39000 g for 20 min. Purified virus formed an opalescent halo in the 50% sucrose layer. The purified virus was collected and diluted with 2 volumes of TMN buffer and centrifuged at 35 000 g for 60 min. The pellet was resuspended in 50 μl of TMN buffer and stored at—80 °C.

### Genomic DNA preparation and sequencing

DNA extraction was performed according to the manufacturer’s instructions of QIAamp DNA mini Kit (QIAGEN). DNASeq library compatible with short read Illumina sequencing was generated using the NEB Ultra DNA library Kit (NEB) starting with 500 ng of DNA, as measured by Qubit (Invitrogen) and following the manufacturer’s instructions. Briefly, DNA was fragmented, end-repaired and subsequently the adapter was ligated. Agencourt AMPure XP beads were used to size select the DNA fragments containing the adapters. Finally, the library was amplified by 15 PCR cycles. The fragment size distribution of the library was analysed on a BioAnalyzer High Sensitivity LabChip showing a size range between 400 and 446 bp with the main peak of the library at 401 bp. The library was diluted to 2 nM and multiplex-sequenced together with five samples on the Illumina MiSeq (2 × 250 bp paired end run, estimated 4.3 million reads/sample).

### Raw sequence data processing, mapping, assembly, and genome annotations

For each sample, the sequenced raw data were processed to obtain high-quality reads. Reads with a quality score of Q ≤ 30 or length under 50 bp were discarded and adapter-trimmed using BBDuk (Bushnell 2019). Sequences were mapped against the host genome (*Ovis aries*) to remove host DNA contamination. Unmapped reads were used as input data for *de novo* assembly using SPAdes genome assembler v3.15.2 (Bankevich et al. 2012). This procedure rendered two (NAV) or three (HRE, CHB, and ARA) contigs that corresponded to ORFV virus according to Blastx. The resulting contigs were aligned to the reference genome NZ2 (DQ184476.1) and manually checked to obtain draft genomes. Finally, each set of high-quality reads was aligned to the corresponding draft genome using Bowtie2. The consensus genome sequences were extracted using bcftools (Danecek et al. 2021). New ORFV whole sequenced genomes were annotated using GATU with NZ2 as genome reference to capture all the potential open reading frames (ORFs) (Tcherepanov et al. 2006).

### Detection of simple sequence repeats

Simple and compound microsatellites were extracted with IMEx software (Mudunuri et al. 2010), which identified perfect mono-, di-, tri-, tetra-, penta-, and hexanucleotide repeats along the genomes characterized. The minimum numbers of iterations were 6, 3, 3, 3, 3, and 3 for mono- to hexanucleotide motifs, respectively, using the parameters previously used for RNA (Chen et al. 2009) and DNA viruses (Wu et al. 2014, Hatcher et al. 2015). Maximum distance allowed between any two SSRs (dMAX) was 10 nucleotides. Other parameters were used as default. Compound microsatellites (cSSR) were not standardized in order to determine real composition.

### Phylogenetic analysis

The whole genome sequences obtained were aligned using the MAFFT version 7 package (Katoh and Standley 2013) and curated manually. The phylogenetic analysis was carried out through the maximum likelihood method using IQ-Tree program version 1.4.2(Minh et al. 2020). IQ-Tree was also used for estimating the substitution model by means of ModelFinder. Then, the tree was built using GTR + G4 evolution model within 000 replicates for bootstrap, and the results were visualized with FigTree 1.4.0 tool (available at https://github.com/rambaut/figtree/releases).

Alternatively, MrBayes version 3.2.7 (Ronquist et al. 2012) was used for building trees through Bayesian methodology, setting nst: 6, rates: gamma, and invariant sites as model parameters. This analysis was run for 10 million Markov chain Monte Carlo (MCMC) iterations, sampling trees every 1000 generations. About 10% of the burn-in was considered. Tracer 1.7 version (Rambaut et al. 2018) was used for checking the convergence and evaluating that the effective sample size for relevant parameters was >200. Finally, the tree topology was visualized with FigTree.

Sequence clusters were determined using the Fast hierarchical Bayesian analysis of population structure algorithm (fastbaps), which applies the hierarchical Bayesian clustering (BHC) algorithm to determine clusters of multi-sequence genotypes (Tonkin-Hill et al. 2019). Based on the obtained alignment, 32 genes were selected based on high sequence conservation among available strains.

### Detection of recombinant regions

The whole genome multiple sequence alignment was analysed in RDP5 software v. 5.5 (Martin et al. 2021). This software applies several analysis methods to identify the presence of recombinant sequences. Data were analysed using the following recombination methods: RDP, GENECONV, Bootscan/Recscan, MaxChi, Chimaera, SiScan, and 3-seq. Recombination events considered were detected by at least 5 of these 7 methods. A recombination breakpoint graph was obtained and used to detect recombinant regions.

### Evolutionary rate and most recent common ancestor estimations

To verify the temporal signal and molecular clocklike behaviour, the TempEst software was implemented (available at https://beast.community/tempest) providing dates of each sample. Evolutionary rate and time to the most recent common ancestor (TMRCA) were estimated through Bayesian coalescent analysis using BEAST program 10.4 version (available at https://beast.community). For this study, a non-parametric Bayesian Skyride coalescent model with an uncorrelated lognormal relaxed clock was used. This analysis was run on samples from the cluster 3, three times for 100 million MCMC iterations each with 10% burn-in, and Tracer 1.7 v. was used to check convergence evaluating that the effective sample size was > 200 for relevant parameters.

## Results

### Genome characterization

The assembled genomes of ARA, CHB, HRE, and NAV were 137 891, 137 160, 137 340, and 137 214 bp long respectively. As expected, all four genomes contained a large central coding region surrounded by two inverted terminal repeat (ITR) regions. In each case, the left end nucleotide was designated as base 1. Aligned with other whole genome sequences of ORFV strains (Table 1), the ARA strain showed the highest similarity with SY17 strain, showing 98.7% nucleotide identity. For CHB and HRE the largest nucleotide identity was observed with NZ2, with 99.1% and 99.0% respectively. NAV strain showed an identity of 99.1% with the TVL strain.

The ITRs of these viruses were 2 943, 3 108, 3 161, and 3 177 bp (ARA, CHB, HRE, and NAV). The ITR of ARA contained the terminal *BamHI* sites and the telomere resolution sequence at both ends, similar to SJ1, GO, YX, MP, NA17, SY17, and CL18 strains. However, CHB, HRE, and NAV strains only contained one *BamHI* site and the conserved telomere-related sequence at the right end, as the NA1 and NP strains.

**Figure 1:**
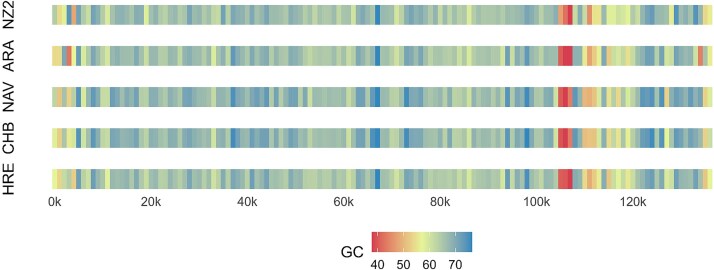
Genome characterization of the full-length genomes. G/C content genome profile of Argentinian (HRE and CHB) and Spanish (ARA and NAV) together with reference strain ORFV-NZ2 (sliding window size: 100 bp; made with in house R script).

The G + C nucleotide composition of these ORFV genomes was ARA 63.89%, CHB 64.22%, HRE 64.31%, NAV 64.32% which are similar to the average value of PPV genomes. We used a moving average analysis with a 1 000 bp window to analyse the G + C percentage from the genomes of the four ORFV isolates using NZ2 as a comparison (Fig. 1). The G + C percentage of each strain was predominantly high in the central region and lower in the terminal regions of the genome. Around positions 103–108 kb, a pronounced deviation from the average G + C content was observed. This recognizable signature can be seen in the other strains used in this work (Supplementary Fig. 1).

**Figure 2:**
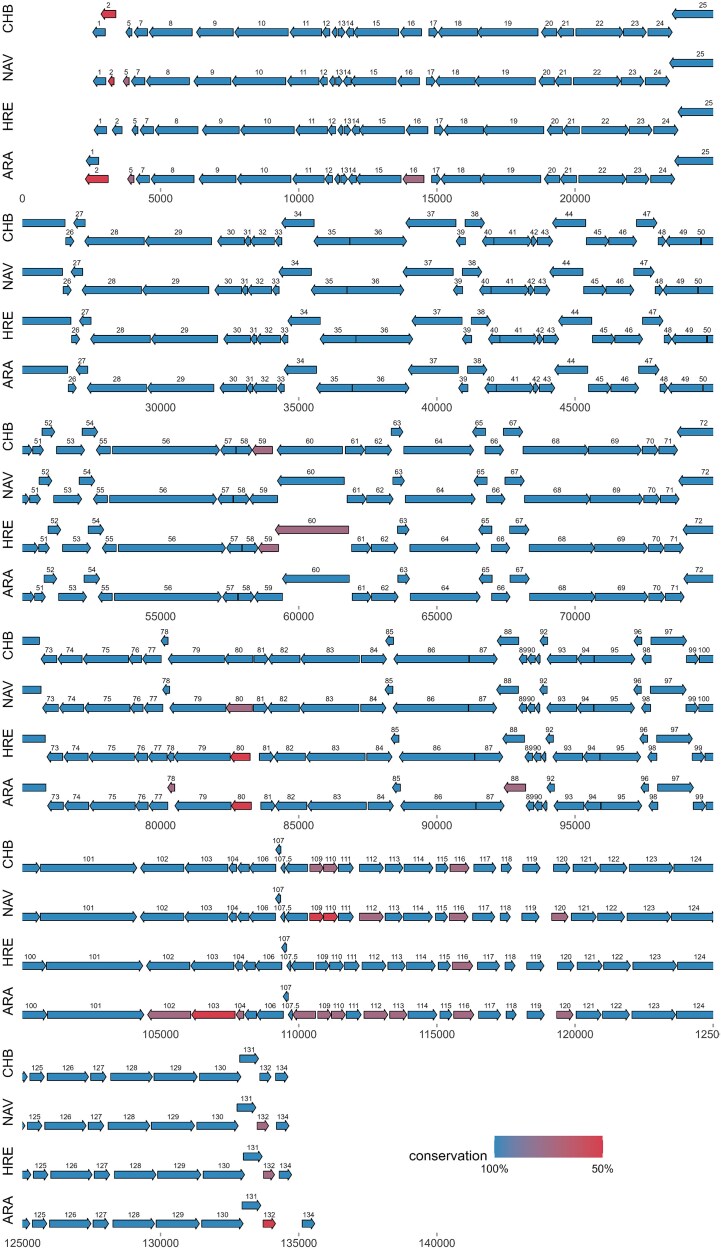
Comparative map of the CHB, HRE, ARA and NAV whole genomes. Each ORF is represented by an arrow that indicates the size and direction of transcription; open arrowheads indicate that an ORF is split over two lines of the diagram. Different colours of arrows indicate amino acid identity of predicted proteins between the corresponding genome and ORFV-NZ2.

Gene annotation was performed using NZ2 as the reference genome, and 132 genes were identified for each sample in this study (Fig. 2). Additionally, genes were predicted based on their localization within the genome, the size of the predicted proteins and by similarity with proteins previously described in PPV. The majority of the open reading frames were non-overlapping, consistent with other poxvirus genomes and showed both, a relatively high amino acid identity and similar length with their NZ2 counterparts (Table 2).

Interestingly, few notable exceptions were found in the genomes described in this work, regardless of geographical origin:

1. ORF002 from ARA, which overlaps with ORF001 and is ~50% larger than the encoded in NZ2.
2. The ORFs 102, 103, and 104 from ARA showed a lower level of identity with NZ2 (80, 65, and 88, respectively) than the other three isolates.
3. Meanwhile, the ORFs 109 and 110 showed lower identity in NAV isolate (64.1 and 64.4%) and somewhat higher (from 85.8%) for ARA and CHB isolates.

### S‌SR and cSSR analysis

The SSR analysis of the four isolates show a Relative Abundance (RA) and a Relative Density (RD) similar to that of NZ2 (Table 3). In each case the most abundant type of repeat was the dinucleotide (DI) followed by TRI, MONO, HEXA, and TETRA repeats. No PENTA repeat was detected.

**Table 2:** Open reading frames (ORFs) of CHB, HRE, NAV and ARA strains of Orf virus compared with the corresponding ORFs of NZ2 strain.

ORF	CHB		HRE		NAV		ARA		Predicted function
	Length (aa)	% Id^*^	Length (aa)	% Id^*^	Length (aa)	% Id^*^	Length (aa)	% Id^*^	
001	149	98.7	149	99.3	149	99.3	149	96.6	Hypothetical protein
002	178	51.9	117	99.1	67	56.4	274	30.3	Hypothetical protein
005	72	95.8	71	100.0	70	91.5	75	88.2	Hypothetical protein
007	160	96.2	159	99.4	160	95.6	159	96.9	Dutpase
008	516	99.2	516	99.6	516	98.6	516	97.5	Ankyrin/F-box protein
009	442	99.8	442	99.3	442	99.5	442	99.3	Hypothetical protein
010	643	99.1	643	99.4	643	99.4	643	98.6	EEV-maturation protein
011	378	99.5	378	100.0	378	100.0	378	100.0	EEV phospholipase
012	89	96.6	89	97.8	89	96.6	89	97.8	Hypothetical protein
012.5	60	100.0	60	100.0	60	100.0	60	98.3	Hypothetical protein
013	79	100.0	79	97.5	79	100.0	79	98.7	Hypothetical protein
014	93	100.0	93	100.0	93	98.9	93	97.8	RING-H2 motif protein
015	539	99.6	539	99.3	539	99.3	539	98.5	Hypothetical protein
016	252	95.4	259	98.1	259	98.1	252	92.7	Hypothetical protein
017	105	100.0	105	100.0	105	100.0	105	97.1	DNA binding phosophoprotein
018	472	99.4	472	99.8	472	99.6	472	99.4	Poly-A polymerase catalytic subunit
019	725	99.2	725	99.3	724	99.3	725	99.2	Hypothetical protein
020	183	98.4	183	98.4	183	97.8	183	98.4	Dsrna-binding, interferon resistance
021	193	99.5	193	99.5	193	99.5	193	99.5	RNA polymerase subunit RPO30
022	567	100.0	567	99.6	567	99.8	567	99.5	Hypothetical protein
023	272	100.0	272	100.0	272	100.0	272	100.0	Membrane protein
024	292	96.9	292	95.9	291	96.6	288	96.5	Hypothetical protein
025	1 008	99.9	1 008	99.7	1 008	99.9	1 008	99.8	DNA polymerase
026	96	100.0	96	100.0	96	100.0	96	100.0	ERV/ALR-like protein
027	137	99.3	137	100.0	137	100.0	137	98.5	Virion core protein
028	715	98.7	719	98.5	713	99.7	713	98.3	Hypothetical protein
029	797	99.9	797	100.0	797	99.9	797	99.1	Hypothetical protein
030	321	100.0	321	100.0	321	100.0	321	99.7	Virion core protein
031	70	100.0	70	100.0	70	100.0	70	100.0	Hypothetical protein
032	285	98.6	285	98.9	285	98.2	286	96.2	DNA binding phosphoprotein
033	78	100.0	78	100.0	78	98.7	78	100.0	IMV protein
034	389	100.0	389	100.0	389	99.7	389	100.0	Telomere binding protein
035	430	100.0	430	100.0	430	100.0	430	99.8	Virion core protease
036	683	99.7	683	99.9	683	99.9	683	99.6	RNA helicase
037	603	99.8	603	99.8	603	99.7	603	99.2	Zn-protease, virion morphogenesis
039	110	99.1	110	98.2	110	100.0	110	97.3	Hypothetical protein
038	231	99.6	231	98.7	231	99.6	231	98.7	Late transcription factor elongation factor
040	137	100.0	137	99.3	137	98.5	137	98.5	Glutaredoxin-like protein
041	452	100.0	451	99.8	452	100.0	448	98.0	Hypothetical protein
042	63	100.0	63	100.0	63	100.0	63	100.0	RNA polymerase subunit RPO7
043	185	100.0	185	100.0	185	100.0	185	99.5	Hypothetical protein
044	398	99.7	398	99.7	398	99.5	397	99.7	Virion core protein
045	266	100.0	266	100.0	266	100.0	266	100.0	Late transcription factor
046	334	99.7	334	99.1	334	99.1	334	97.3	Myristylprotein
047	244	100.0	244	100.0	244	100.0	244	99.6	IMV protein
048	90	100.0	90	100.0	90	100.0	90	100.0	Hypothetical protein
049	418	98.6	418	98.8	417	98.6	417	98.3	Hypothetical protein
050	259	99.6	259	98.8	259	99.2	259	99.2	Virion core protein
051	128	98.4	128	99.2	128	99.2	128	98.4	Membrane protein
052	151	99.3	151	99.3	151	100.0	151	100.0	Virion protein
053	336	100.0	336	99.7	336	100.0	336	100.0	Poly(A) polymerase subunit
054	186	100.0	186	100.0	186	100.0	186	98.9	RNA polymerase subunit RPO22
055	167	99.4	167	99.4	167	99.4	167	98.8	Late membrane protein
056	1 289	100.0	1 289	100.0	1 289	100.0	1 289	99.9	RNA polymerase subunit RPO147
057	181	100.0	181	99.4	181	99.4	181	99.4	Tyrosine phosphatase, virus assembly
058	191	100.0	191	100.0	191	100.0	191	100.0	IMV, viral entry
059	241	71.6	241	71.6	336	99.4	340	97.4	Immunodominant envelope protein
060	786	96.3	883	87.9	804	100.0	804	99.6	RNA-polymerase associated RAP94
061	229	98.3	229	98.3	226	97.8	226	97.4	late transcription factor VLTF4
062	318	100.0	318	100.0	318	100.0	318	100.0	Topoisomerase I
063	138	100.0	138	99.3	138	100.0	138	99.3	Hypothetical protein
064	841	99.9	841	99.9	841	99.8	841	99.8	mRNA capping enzyme subunit
065	156	100.0	156	100.0	156	100.0	156	100.0	Virion protein
066	221	99.5	221	99.5	221	98.6	221	99.5	Virion protein
067	231	99.6	231	99.6	231	100.0	231	99.1	Uracil-DNA glycosylase
068	787	100.0	787	100.0	787	100.0	787	100.0	NTPase
069	635	99.8	635	99.8	635	99.8	635	99.8	Early transcription factor
070	190	99.5	186	97.4	190	99.5	190	99.5	RNA polymerase subunit RPO18
071	224	100.0	224	100.0	224	100.0	224	99.6	NTP pyrophosphohydrolase
072	638	99.8	638	99.8	638	99.7	638	99.8	NPH-1
073	188	98.9	188	98.9	188	99.5	188	98.4	Hypothetical protein
074	289	100.0	289	100.0	289	100.0	289	99.7	mRNA capping enzyme
075	545	100.0	545	100.0	545	100.0	545	100.0	Rifampicin resistance, membrane protein
076	150	100.0	150	99.3	150	99.3	150	98.7	Late transcription factor VLTF2
077	224	100.0	224	100.0	224	100.0	224	99.6	Late transcription factor VLTF3
078	82	98.8	79	95.1	82	97.6	83	90.4	Thioredoxin-like protein
079	675	99.9	674	99.4	675	99.4	675	99.4	virion core, P4b precursor
080	339	98.3	339	96.2	339	99.1	339	94.3	Virion core protein
081	172	99.4	172	98.3	172	99.4	172	99.4	RNA-polymerase subunit RPO19
082	378	99.2	378	98.4	378	99.2	378	98.1	Hypothetical protein
083	706	99.9	706	99.9	706	99.7	706	99.9	Early transcription factor
084	303	100.0	303	100.0	303	100.0	303	99.7	Intermediate transcription factor
085	93	100.0	93	100.0	93	100.0	93	100.0	Virion membrane protein
086	905	99.8	905	99.9	905	99.8	905	99.2	Virion core protein P4a precursor
087	336	100.0	336	100.0	336	100.0	336	100.0	Virion formation
088	258	95.4	261	97.0	261	98.5	258	94.7	Virion core protein
089	92	98.9	92	100.0	92	98.9	92	100.0	Virion membrane protein
090	91	100.0	91	98.9	91	100.0	91	98.9	IMV membrane protein
091	53	100.0	53	100.0	53	100.0	53	98.1	Putative virulence factor, IMV
092	89	100.0	89	100.0	89	100.0	89	100.0	Hypothetical protein
093	358	99.4	358	99.7	358	99.7	358	98.9	Myristylated protein
094	196	100.0	196	100.0	196	99.5	196	98.0	Phosphorylated IMV membrane protein
095	488	99.8	488	99.8	488	100.0	488	99.8	DNA helicase
096	91	100.0	91	98.9	90	97.8	91	100.0	Zn-finger protein
098	108	99.1	108	100.0	108	100.0	108	100.0	Hypothetical protein
097	429	99.8	429	99.8	429	99.8	429	99.3	DNA polymerase processivity factor
099	146	100.0	146	100.0	146	100.0	146	100.0	Resolvase
100	377	99.2	380	99.7	380	100.0	380	99.7	Intermediate transcription factor VITF3
101	1 161	100.0	1 161	100.0	1 161	100.0	1 161	100.0	RNA polymerase RPO132
102	520	99.8	520	98.7	520	98.8	518	80.7	A-type inclusion protein/fusion peptide hybrid
103	516	99.6	516	97.1	516	96.7	522	65.2	A-type inclusion protein
104	90	95.6	90	97.8	90	97.8	90	87.8	Viral fusion peptide
105	140	99.3	140	99.3	140	99.3	140	99.3	IMV surface protein
106	314	99.7	314	99.4	314	99.7	317	98.4	RNA polymerase subunit RPO35
107	60	100.0	60	100.0	60	100.0	60	100.0	Virion morphogenesis
107.5	49	95.9	49	95.9	49	98.0	49	95.9	Hypothetical protein
108	266	98.9	266	98.9	266	98.9	271	94.9	Atpase, DNA packaging
109	160	85.8	160	98.1	165	64.1	160	87.7	EEV glycoprotein
110	165	90.3	165	100.0	167	64.4	165	93.3	EEV glycoprotein
111	179	99.4	179	99.4	179	98.9	179	98.9	Hypothetical protein
112	287	96.9	287	96.9	286	92.7	286	92.3	Chemokine binding protein
113	211	95.7	208	97.1	206	95.2	205	94.3	Hypothetical protein
114	346	98.6	346	98.8	346	98.8	346	97.4	Hypothetical protein
115	145	99.3	145	97.9	143	96.6	143	95.2	Hypothetical protein
116	228	92.7	244	87.8	223	91.0	240	86.2	Hypothetical protein
117	265	99.2	265	98.9	265	99.6	265	98.9	GM-CSF/IL-2 inhibition factor
118	119	98.3	119	98.3	119	100.0	119	97.5	Hypothetical protein
119	206	98.1	206	98.1	206	98.5	206	96.6	Hypothetical protein
120	196	97.5	196	97.0	196	93.1	195	93.5	Hypothetical protein
121	306	99.3	300	97.7	300	97.4	300	95.1	Hypothetical protein
122	323	99.1	323	99.4	323	99.1	323	97.5	Hypothetical protein
123	525	98.7	525	99.4	525	99.0	525	98.5	Ankyrin/F-box protein
124	532	99.1	532	99.1	532	99.4	532	97.7	Hypothetical protein
125	173	99.4	173	99.4	173	98.8	173	98.8	Hypothetical protein

*(continued)*

**Figure 3:**
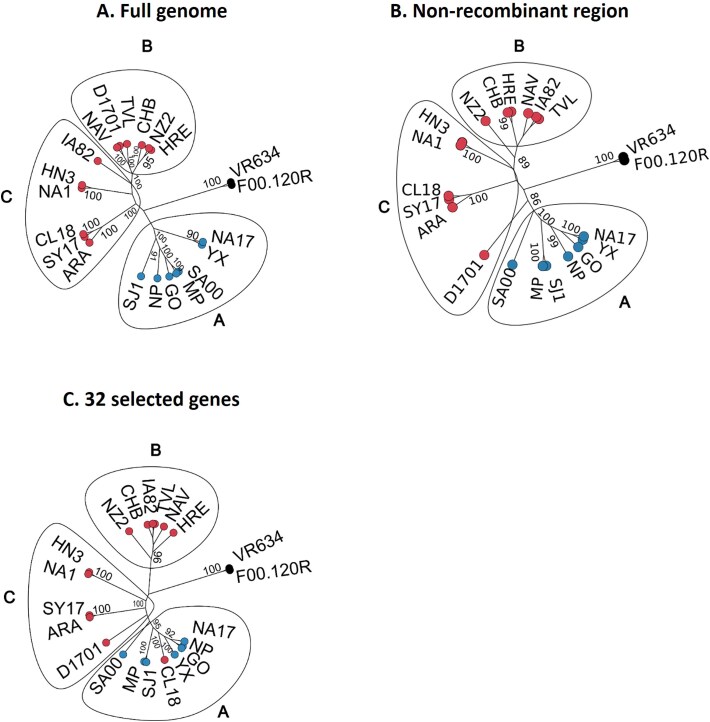
Comparative map of the CHB, HRE, ARA and NAV whole genomes. Each ORF is represented by an arrow that indicates the size and direction of transcription; open arrowheads indicate that an orf is split over two lines of the diagram. Different colours of arrows indicate amino acid identity of predicted proteins between the corresponding genome and ORFV-NZ2.

Additionally, the compound SSR (cSSR) composition was obtained and the occurrence of each cSSR in the 50 PPV full genomes used in this work was analysed. Of the 308 cSSR found, more than half (159) were present in only one sample each. Furthermore, 48 ORFV complete sequences presented the cSSR (GC)3-x1-(AC)3, that was not present in PCPV samples (F00.120R and VR634). Interestingly, cluster 1 and cluster 2 presented 3 and 1 specific cSSR, respectively (Table 4 and Fig. 3).

### Phylogenetic analysis

**Figure 4:**
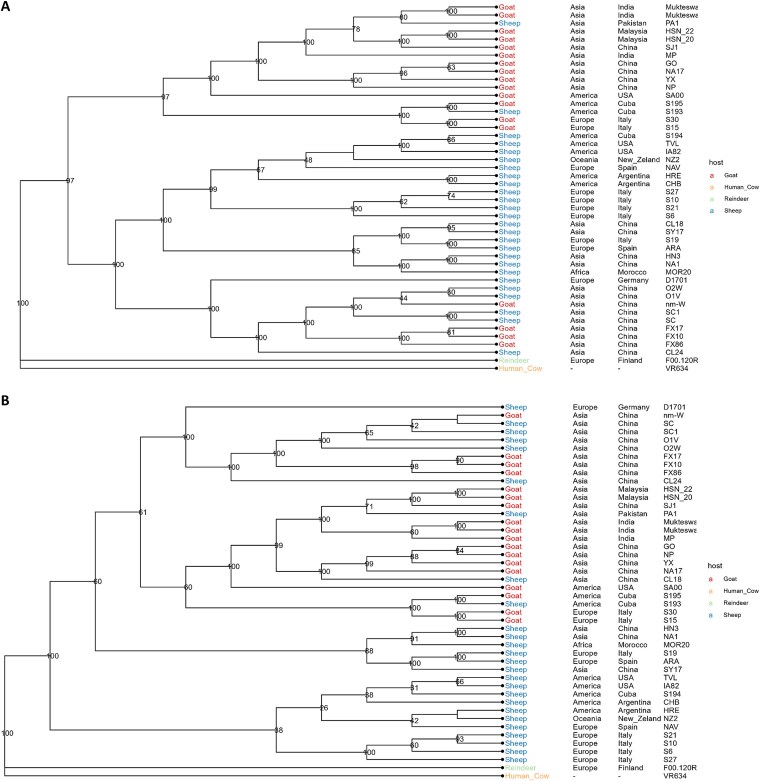
Phylogenetic analysis. Maximum-likelihood phylogenetic tree based on genome sequences, excluding recombinants regions (A), and 32 concatenated selected genes (B). The numbers at nodes correspond to bootstrap values. Tip labels indicate goat and sheep samples, respectively.

The phylogenetic analysis was performed at three different levels: full genome, excluding recombinant regions and using 32 selected genes (Fig. 3, Fig. 4 and supplementary Fig. 2).

The phylogenetic tree derived from the full-length genome alignment was well supported, with a group structure of three clusters, namely 1, 2, and 3 (Fig. 3).

Cluster 1 is composed mostly by goat and sheep sequences from Chinese origin, with the exceptions of D1701 sample from Germany. Cluster 2 does not seem to have a monophyletic origin and is formed mostly by goat strains, which share the (CG)4-X0-(GC)3 cSSR. Interestingly, only Asian strains of this group presented the molecular signature of the (CT)3-X7-(CG)3 cSSR (Fig. 3). Cluster 3 comprised sheep strains, with representation from North, Central, and South America, Europe, Africa, and only four Chinese strains. The four strains in this study (ARA, CHB, HRE, NAV) belong to cluster 3. Interestingly, sequences from wild ruminants and a human strain are placed within cluster 3 (Supplementary Figure 3).

In order to test if a better separation between species could be achieved, by avoiding tne inclusion of recombination areas that might confuse the phylogenetic signal, recombinant regions were identified and excluded from the alignment (positions 19 000 to 100 904 of the full-length genome alignment were retained). A concatenated sequence 81 905 bp-long was obtained for each sample. The obtained tree showed a similar group structure with the three clusters (Fig. 4), but now it is possible to establish better phylogenetic relationships in cluster 2. In cluster 3, two groups emerged: one containing Argentine strains CHB and HRE and Spanish NAV along with strains from Cuba, the United States, and Italy; while in the other group we can find the Spanish ARA strain along with strains from China, Morocco, and the Italian S19.

**Table 2:** Continued.

ORF	CHB		HRE		NAV		ARA		Predicted function
	Length (aa)	% Id^*^	Length (aa)	% Id^*^	Length (aa)	% Id^*^	Length (aa)	% Id^*^	
126	497	99.6	497	99.8	497	99.2	497	99.6	Ankyrin/F-box protein
127	186	97.8	185	96.2	186	97.3	185	95.7	Interleukin 10
128	501	98.4	501	98.8	501	98.4	501	96.4	Ankyrin/F-box protein
129	520	99.2	520	99.2	520	98.8	520	99.2	Ankyrin/F-box protein
130	498	100.0	498	99.8	498	100.0	497	99.8	Protein kinase
131	225	99.6	225	99.1	225	99.1	225	96.5	Membrane protein
132	133	97.0	132	89.5	137	80.9	148	54.2	vegf-e
134	149	98.7	149	99.3	149	99.3	149	96.6	Hypothetical protein

^*^The amino acid identity corresponding with NZ2 (GenBank accession number: DQ184476.1)

**Table 3:** Open reading frames (ORFs) of CHB, HRE, NAV and ARA strains of Orf virus compared with the corresponding ORFs of NZ2 strain.

SAMPLE	G + C (%)	SSR	RA	RD	MONO	DI	TRI	TETRA	PENTA	HEXA	SSR coding	SSR non-coding	cSSR	cSSR-coding
ARA	83.25	934	6.77	48.22	5.35	75.16	19.06	0.21	0	0.21	90.69	9.31	64	93.75
NAV	84.00	946	6.89	49.32	5.39	74.84	19.24	0.21	0	0.32	90.80	9.20	67	89.55
CHB	83.01	951	6.93	49.35	5.47	75.50	18.61	0.00	0	0.42	90.64	9.36	64	87.50
HRE	84.15	936	6.82	48.78	5.34	74.68	19.34	0.32	0	0.32	91.77	8.23	63	93.65
NZ2	83.54	954	6.92	49.64	5.24	74.84	19.08	0.31	0	0.52	90.67	9.33	68	86.76
SA00	80.64	1 012	7.23	51.96	4.94	74.51	18.28	2.08	0	0.20	85.08	14.92	75	82.67

SSR. The counts of microsatellites in each genome.

RA. Relative abundance of microsatellites (number of microsatellites or compound microsatellites/kb).

RD. Relative density of microsatellites is defined as the total length (bp) contributed by each microsatellite per kb of sequence.

MONO, DI, TETRA, PENTA and HEXA are the percentage of each type of repeat in each genome.

SSR coding and SSR non-coding are the percentage of SSR in genic and intergenic regions, respectively, for each genome.

cSSR. The counts of compound microsatellites in each genome.

**Table 4:** Compound simple sequence repeats (cSSR) relevant for ORFV whole genome cluster classification.

cSSR	Cluster 1	Cluster 2	Cluster 3	F00.120R and VR634	Protein	Hypothetical function	sequence
(GC)3-x1-(AC)3	x	x	x	-	ORFV081	RNA-polymerase subunit RPO19	gcgcgctacacac
(CG)4-x0-(GC)3		x	-	-	ORFV038	Late transcription factor elongation factor	cgcgcgcgcggcgcgc
AGG)3-x7-(GC)3	x	-	-	-	ORFV064	mRNA capping enzyme subunit	aggaggagggcaagttgcgcgc
(TC)3-x7-(C)7-x6-(CG)3	x	-	-	-	ORFV017	DNA binding phosphoprotein	tctctcccgccgtcccccccatgccccgcgcg
(TTCC)3-x-3-(TCC)3	x	-	-	-	-	-	ttccttccttcctcctcc
(GC)3-x4-(GC)3	x	-	-	-	ORFV128	Ankyrin/F-box protein	gcgcgcccgagcgcgc
(CT)3-x7-(CG)3	-	Partial	-	-	ORFV063	Hypothetical protein	ctctctgcgcacacgcgcg

cSSR. The relevant compound SSR. Clusters are those that correspond to whole genome dataset. The specific sequence is shown for each cSSR.

Moreover, we selected 32 highly conserved genes for the family Poxviridae (Babkin and Babkina 2011; Le Loc’h et al. 2015), all present in the dataset excluding recombinant regions. Most of these genes encode proteins involved in viral DNA replication and transcription. Specifically**,** a 42 417 bp-long concatenated sequence was identified and the obtained tree was well supported (bootstrap value of 100%). The maximum likelihood tree showed a similar group structure consistent with previous analyses (Fig. 4B).

### Evolutionary estimated rate and most recent common ancestor

We performed a molecular clock analysis on all three datasets obtaining well-supported Bayesian trees (posterior probability of 1), confirming clade composition and group structure observed with the maximum-likelihood method (data not shown). No convergence was obtained when the full-length genome or the non-recombinant regions datasets were considered. But convergence was obtained for the 32 selected genes dataset.

The temporal signal analysis exhibited a positive correlation between genetic divergence and time, however diffuse regression plots (R^2^ = 0.043) were obtained using the full-length genome dataset (Supplementary File 1). Analysing only cluster C, a slight improvement in the regression coefficient was obtained (R^2^ = 0.099). These results strongly suggest an uneven evolutionary rate among the branches, so a relaxed molecular clock was chosen for the next phylogenetic analysis.

The MCMC analysis, performed with Beast software for the worldwide cluster 3 that includes the four samples described in this study, estimated a nucleotide substitution rate of 2.6 x10^−5^ subs/site/year (9.2x10^−8^—6.7x10^−5^—95% HPD) placing the TMRCA (median divergence time) around 1729 (X—95% HPD). The result was consistent among the three runs.

## Discussion

In this work, we isolated and sequenced four novel ORFV strains named ARA, CHB, HRE, and NAV from infected sheep in Argentina and Spain. The whole genomic sequence of the isolates ranged between 137 160 and 137 891 bp, including the inverted terminal repeats. Despite differences in length, all the sequences obtained resembled those publicly available in gene composition, encoding 132 non-overlapping genes as featured by other ORFV.

The G + C content for all samples was ~64%. This value coincides with that described for other genomes of the PPV genus (Mercer et al. 2006). A small region located approximately between 103 and 108 kb with a low G + C concentration (40%–45%) draws attention. This coincides with previous findings in ORFV genomes, and also in PCPV and BPSV genomes, so it could be considered a molecular signature of the PPV genus as previously proposed (Mercer et al. 2006; Hautaniemi et al. 2010).

Characterization of the four genomes with respect to microsatellites showed between 934 and 951 SSRs per genome, with RA and RD values between 6.77–6.89 and 48.22–49.35 respectively. These values are slightly lower than those reported by Sahu et al. 2020, but within the ranges found for other DNA viruses (Ouyang *et al.* 2012, Singh *et al.* 2014). In the four genomes, the most abundant repetitive units were: dinucleotide (75.04%), trinucleotide (19.06%) and mononucleotide (5.38%). The majority of the SSRs and cSSRs were found in coding regions (90.97% and 91.11% respectively). This distribution is reasonable since viruses have short intergenic regions and can often overlap with coding regions.

Certain studies have suggested the use of SSR and cSSR for the characterization of viral strains (Houng et al. 2009; Burrel et al. 2013). We focused on the analysis of cSSRs since, given the complexity of their structure, they could serve as specific spots to determine relationships between viral isolates. Cluster 1, primarily consisting of Chinese sheep and goat strains, shares three cSSRs: (TTCC)3-X3-(TCC)3, (AGG)3-X7-(GC)3 and (TC)3-X7-C7-X6-(CG)3. The only exception to this molecular signature is the German strain D1701, which lacks any of these cSSRs. This strain was obtained after multiple passages in cell culture and is characterized as attenuated due to the loss of genic regions (Cottone *et al.* 1998), which could explain the partial or complete absence of these cSSRs.

Cluster 2 (mainly sheep strains) presented a specific cSSR (CG)4-(GC)3 but only Asian samples contained the cSSR (CT)3-X7-(CG)3 (Fig. 3). It was not possible to find a cSSR as a marker for group 3. Maintaining the structure of cSSRs is complex because different forces act: mutations, duplication, recombination, *etc.* It is possible that the members of group 3 have an incomplete cSSR and therefore cannot be detected by standard microsatellite studies (Delgrange and Rivals 2004, Mudunuri and Nagarajaram 2007). Having information from more genomes would allow us to determine if these cSSRs are really a molecular signature of groups 1 and 2 or if they are a residual in the differentiation of these groups.

Our phylogenetic analyses with full-length genomes (Fig. 3), and in datasets with partial sequences, based on the whole genome excluding recombinant regions (Fig. 4A) and the 32 selected conserved genes (Fig. 4B) suggest a viral population structuring in three well-supported clusters.

The four samples that originated from sheep and were isolated from Argentina (CHB and HRE) and Spain (NAV and ARA) grouped with other sheep samples (Cluster 3). A phylogenetic analysis based on the whole genomic sequences of 44 ORFV strains revealed that CHB and HRE strains isolated from Argentinian sheep have a close relationship with NZ2 from New Zealand. Spanish sequence NAV was highly similar to TVL and S194 strains (USA and Cuba respectively), while ARA (Spain) was associated to Chinese SY17 and CL18, and MOR20 from Morocco.

Regarding the two Spanish samples sequenced and analysed in this paper, it is remarkable that both belong to two different groups/clades, confirming the existence of at least two genomic clusters circulating in this country.

On the contrary, the two Argentinean samples showed a closer relationship within the same cluster. Nevertheless, previous descriptions have found the presence of distinct clusters in the country (Peralta et al. 2018) based on analysis of partial sequences from genes *orf011*, *orf020*, *orf109*, and *orf127*, so it is essential to gather data from additional sequences in order to accept or reject this hypothesis. In addition to the difference in the data set that was used in both studies, in Peralta et al. 2018, ORFV strains from goats were analysed, so we could be observing a different evolutionary history of the ORFV in Argentina, depending on the host species. However, it is essential to gather data from additional sequences in order to accept or reject this hypothesis.

Interestingly, some research using specific genes (Chi et al. 2015, Coradduzza et al. 2021) has established a clustering of sequences by host. However, when whole genomes are analysed (Coradduzza et al. 2024, and Figs. 3 and 4A of this work), although the phylogenetic tree is separated into two large branches, there are sequences from caprine in the ovine branch and there are sequences from ovine in the caprine branch. Additional analysis including more sequences, ideally from a more representative geographic background, would clarify this point.

Due to the size and complexity of the genome, the analysis using whole genome sequences can generate discordant signals and incompatible results when compared to partial sequence analysis. For this reason, in this work, we conducted a gene selection reducing the interfering signals, bioinformatics cost and offering more reproducible results. Of the 90 genes that form the core of Chordopoxviruses (Upton et al. 2003) we selected 32 genes, many of which were used to determine the molecular clock either in Orthopoxvirus (Babkin and Babkina 2011) or in Avipoxvirus (Le Loc’h et al. 2015). It is not surprising that it was only with this set of data, that convergence was reached and molecular dating of the analysed sequences was obtained.

As far as we know, this is the first molecular epidemiology study that includes ORFV sequences using 32 highly conserved genes and showing comparable results to the full-length genome.

The nucleotide substitution rate inferred in this work was 2.6x10^−5^ subs/site/year. This value could be considered high for a DNA virus, but in accordance with previous works studying Myxoma virus (Kerr et al. 2017) or Avipox viruses (Le Loc’h et al. 2015). Noteworthy, the analysis of MCRA for the worldwide cluster 3, suggested a date in the 18th century, not far from the first case of ORFV reported in a sheep by Steeb in 1787 (Barraviera 2005). However, it is important to include more sequences in the analysis to reduce the confidence interval.

Since sheep and goats are not native animals to South America, it could be hypothesized that the ORFV arrived on the continent with colonization from Europe. The analysis of the four genomes available in our study (two from Spain and two from Argentina) does not allow us to confirm this hypothesis, although the Spanish NAV strain belongs to the same subgroup as the Argentine HRE and CHB. While whole-genome analysis can be challenging, it provides much more information than the analysis of individual genes, where each gene can tell a different evolutionary story (Li et al. 2023).

In this work, we determined the complete genome of two ORFV strains from Spain and two from Argentina. This is the first report for South America and the first for the Iberian Peninsula. Genetic characterization of ORFV strains is the first step towards the development of molecular tools oriented to diagnostics and vaccine development. Furthermore, increasing the knowledge on ORFV strains genetic composition will establish relationships and contribute to future epidemiological studies. Moreover, the ORFV evolutionary analysis will gain precision when more whole genome sequences become available.

## Supplementary Material

Supplementary_materials_veag004_Figure_1

Supplementary_materials_veag004_Figure_2

Supplementary_materials_veag004_File_1

## Data Availability

Whole genome sequences from four Orf virus strains were deposited on the NCBI database (accession numbers: ON805831, ON805830, ON805832 and ON805833). Additionally, already deposited sequences were used: DQ184476, KF234407, MG674916, MG712417, KY053526, HM133903, AY386264, AY386263, KP010356, KP010355, KP010354, MT332357, MN454854, KP010353, GQ329670.1, MN648219.1, GQ329669.1, ON691519, ON691520, ON691521, ON691522, ON691523, ON691524, ON691525, MW537048, OP562382, ON932451, ON932452, PQ374835, PQ374836, PV126639, PP911590.1, PQ685033.1, PP943425.1, PP943426.1, PP943427.1, OR637323.1, OR637324.1, OR637325.1, ON380499.1, ON380500.1, OP151442.1, KF837136.1, SRR9974556, SRR9974557 and SRR9974558
