# Supplementary figures and images for: The whole genome analysis of four Orf virus strains from Europe and South America
Source: Virus Evol. 2026 Jan 28;12(1):veag004. doi: 10.1093/ve/veag004 (PMC12922540; doi:10.1093/ve/veag004)

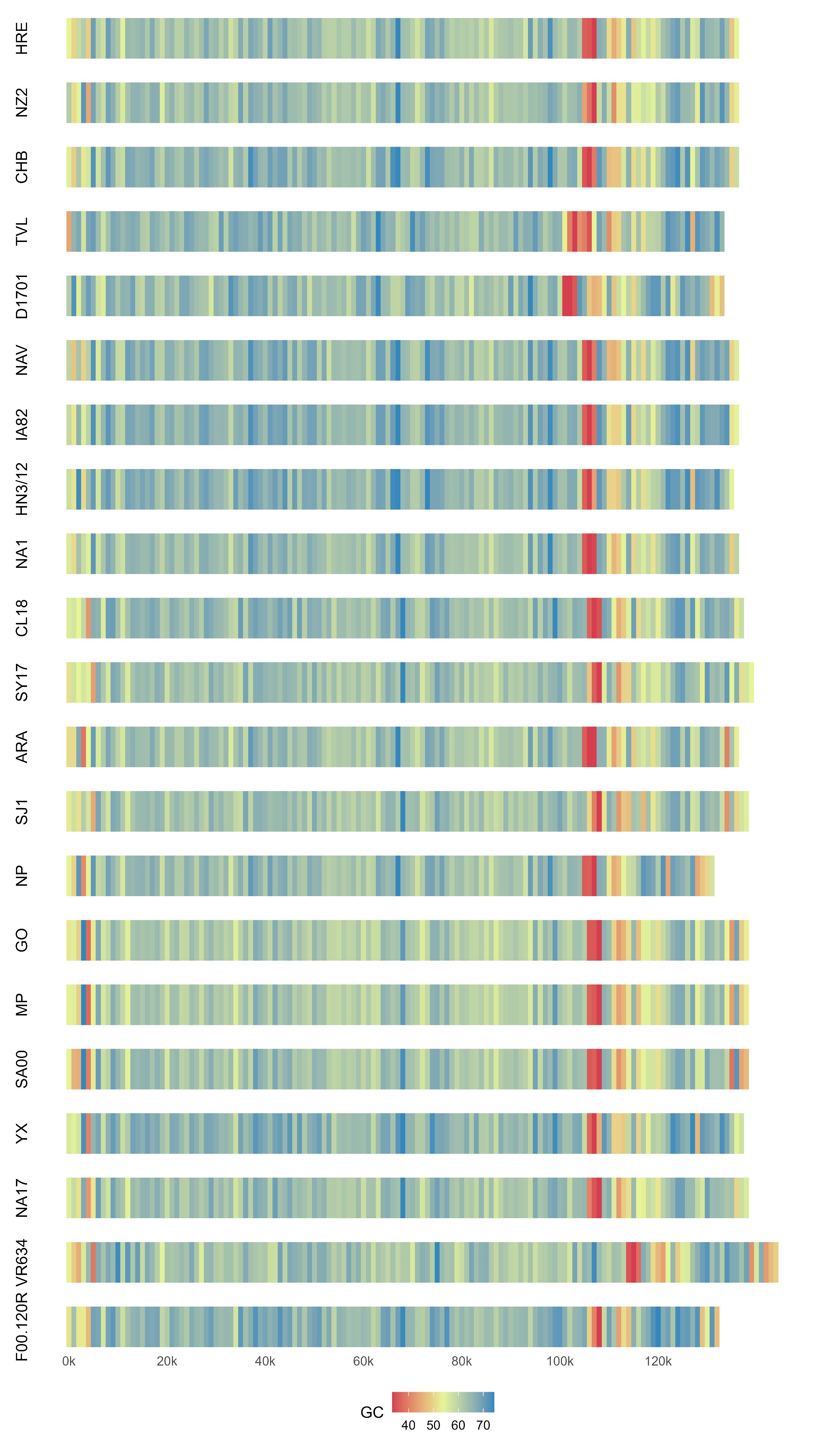

Supplement: Supplementary_materials_veag004_Figure_1 [file supplementary_materials_veag004_figure_1.jpeg]

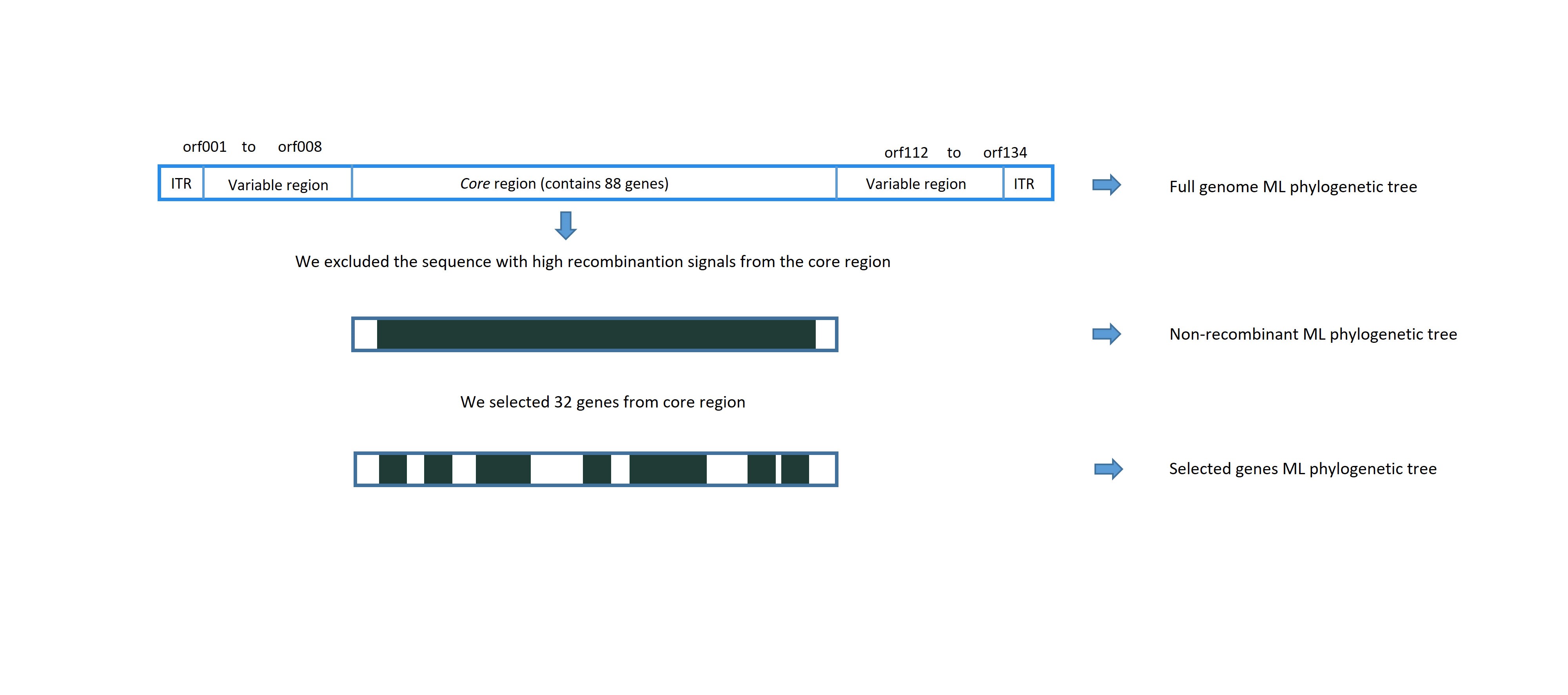

Supplement: Supplementary_materials_veag004_Figure_2 [file supplementary_materials_veag004_figure_2.jpeg]

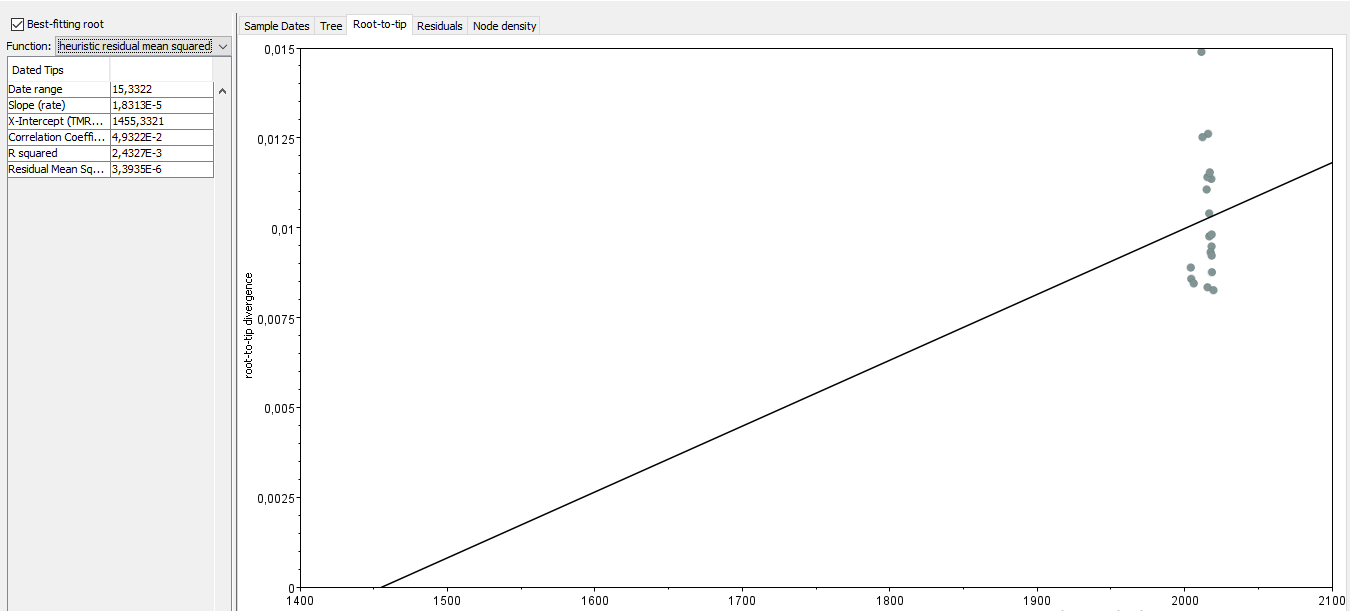
**TEMPEST**

**Combined TRACER**

**AGE_ROOT**


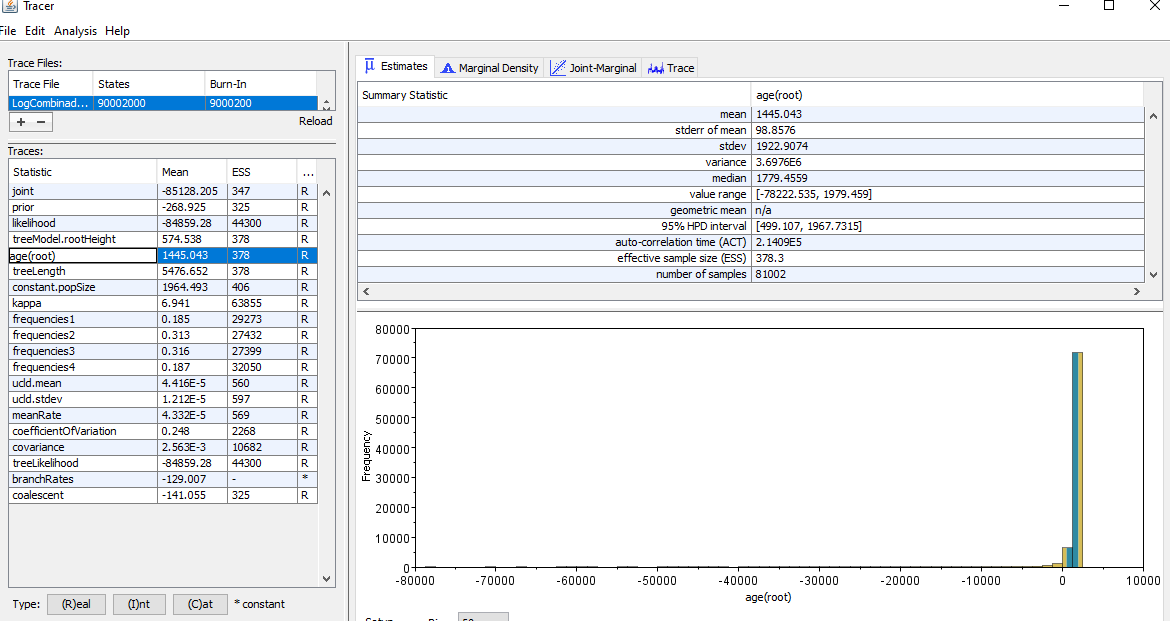


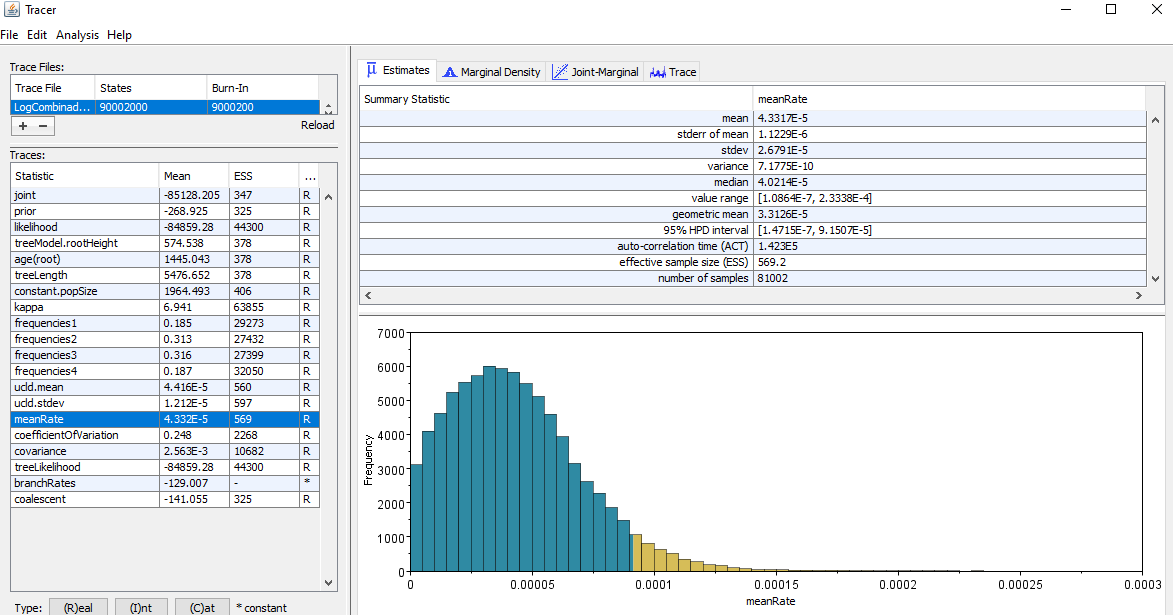
**RATE**

**MCC tree**

**
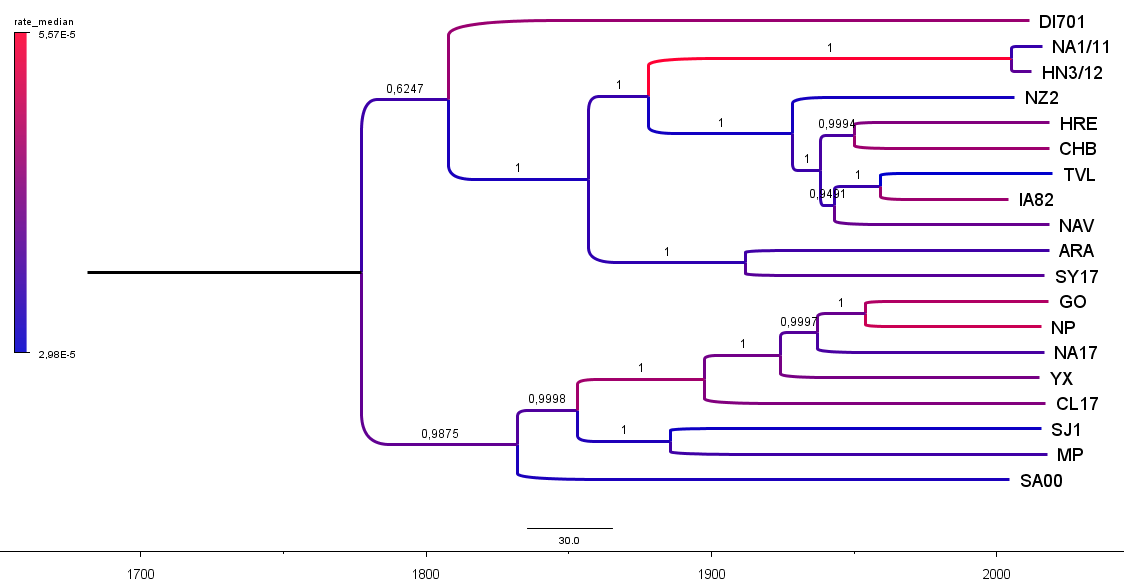
**

Supplement: Supplementary_materials_veag004_File_1 [file supplementary_materials_veag004_file_1.docx]
